# Evaluation of antibiotic prescribing patterns for surgical prophylaxis before and after an educational campaign about penicillin allergies: a single-center, retrospective study

**DOI:** 10.1017/ash.2024.428

**Published:** 2024-10-10

**Authors:** Megan Backus, Marianne Romanos, Anthony Wasielewski, Natalie Paz, Timothy P. Gauthier

**Affiliations:** 1 Resident Pharmacist, Baptist Health Boca Raton Regional Hospital, Boca Raton, FL, USA; 2 Clinical Pharmacy Specialist, Internal Medicine, Baptist Health Boca Raton Regional Hospital, Boca Raton, FL, USA; 3 Clinical Pharmacy Specialist, Infectious Diseases/Antimicrobial Stewardship, Baptist Health Boca Raton Regional Hospital, Boca Raton, FL, USA; 4 Clinical Pharmacist, Baptist Health Boca Raton Regional Hospital, Boca Raton, FL, USA; 5 Antimicrobial Stewardship Clinical Program Manager, Baptist Health, Miami, FL, USA

## Abstract

After an educational campaign about reasonable use of cefazolin for surgical prophylaxis, optimal preoperative antibiotic regimen utilization increased from 52% to 67% (*P* = 0.011). Education to physicians on penicillin allergies may contribute to meaningful increases in use of optimal antibiotics while reducing readmissions and acute care length of stay.

## Introduction

Current evidence suggests approximately 10% of patients have a documented allergy to penicillin, with up to 90% of these cases inaccurately documented.^1^ Patients with a documented penicillin allergy often do not receive first-line antibiotics for surgical prophylaxis due to the misconception that the shared β-lactam ring between cefazolin and penicillin will produce allergic cross-reactivity. Receiving suboptimal antibiotics unnecessarily may contribute to increased risk of adverse drug reactions and undesirable clinical outcomes, such as increased odds of surgical site infections (SSIs).^2,3^

For surgical patients who require antibiotic prophylaxis, cefazolin is the first-line therapy for many surgery types.^4^ Updated literature has shown cross-reactivity is not based on the shared β-lactam ring, but rather related to similarities between the penicillin and cephalosporin R_1_ side chains.^5^ Cefazolin, a first-generation cephalosporin, does not share a similar side chain with any Food and Drug Administration approved β-lactam antibiotic.^6^ This study evaluated the rate of optimal antibiotic regimen utilization for surgical prophylaxis in penicillin allergy-labeled patients before and after a prescriber-facing educational campaign about penicillin allergies.

## Methods

This was a single-center, retrospective cohort study conducted at a tertiary care community hospital. An educational and quality-improvement initiative occurred focusing on improving current antibiotic prescribing practices. Education was provided over one month in-person by infectious diseases and surgical services pharmacists to surgeons and anesthesiologists, including review of an educational handout detailing current penicillin allergy literature, the importance of collecting a complete allergy history, cefazolin side-chain dissimilarity, and when cefazolin should be considered or avoided (Appendix 1). This content was also provided to all pharmacists, presented at the hospital’s surgery committee, emailed to all physicians, and posted to the system intranet.

Patients were identified for study inclusion from a generated report listing patients with a penicillin allergy label who received antibiotics. Screened patients were eligible for inclusion if ≥18 years old and underwent a surgical procedure during the study periods (pre-period of Jan-Dec 2022 and post-period of May 2023–Jan 2024). Patients were excluded if pregnant, incarcerated, had an infection at time of procedure, or received systemic antibiotics within three days prior to the procedure.

The primary outcome was rate of optimal preoperative antibiotic regimen utilization for surgical prophylaxis. Optimal regimens were defined as first-line regimens, or second-line regimens if meeting select allergy criteria. First-line preoperative antibiotic regimens were defined based on antimicrobial prophylaxis in surgery guidelines.^4^ A second-line antibiotic regimen was permissible if the patient’s documented penicillin allergy had a reaction of anaphylaxis, angioedema, bronchospasm, urticaria, hypotension, or swelling. Secondary outcomes included all-cause hospital readmission within 90 days, all-cause mortality within 90 days, SSI rate (following National Healthcare Safety Network definitions), acute care length of stay, allergic reaction rate, preoperative guideline-directed dose selection, and pharmacy clinical intervention rate.

An estimated total sample size of 340 patients, 170 patients each in the pre-education and post-education groups, was required to achieve 80% power to detect a 15% difference between the group proportions. A *P* value of <0.05 was considered statistically significant. Variables were evaluated with χ^2^, Fisher’s exact, or Kruskal-Wallis tests, as appropriate. Analyses were performed using SAS 9.4. This study was approved by the local Institutional Review Board.

## Results

A total of 630 patients were screened. 340 patients were included, evenly split into the pre- and post-groups. The most common reasons for exclusion were no surgical procedure (n = 153) and pregnancy (n = 77). Baseline characteristics for those included were similar between both groups (Table 1). Optimal preoperative antibiotic regimen utilization occurred in 89 patients in the pre-education group (52%) and in 113 patients in the post-education group (67%) (*P* = 0.011). By surgical classification, notable increases in optimal regimen utilization were seen in small intestine hernia repairs (0/5 (0%)–4/9 (44%), *P* = 0.22), hysterectomies (9/19 (47%)–19/24 (79%), *P* = 0.05), and orthopedic surgeries (29/50 (58%)–36/43 (84%), *P* = 0.01).

**Table 1.** Baseline patient characteristics

Patient Characteristic	Pre-Education Group (n = 170)	Post-Education Group (n = 170)	*P* Value
Age, median (IQR), years	71 (63-77)	73 (61–79)	0.34
Sex - Female, n (%)	119 (70)	123 (72)	0.72
Surgery Type, n (%)			0.49
Orthopedic	50 (29)	43 (25)	
Neurosurgery	26 (15)	29 (17)	
Hysterectomy	19 (11)	24 (14)	
Cardiac	14 (8)	19 (11)	
Urologic	12 (7)	4 (2)	
Gynecological	9 (5)	8 (5)	
Small Intestine, Hernia Repair	5 (3)	9 (5)	
Other*	35 (21)	34 (20)	
Surgical Wound Classification, n (%)			0.40
Not Listed	14 (8)	14 (8)	
Class I/Clean	107 (63)	107 (63)	
Class II/Clean-Contaminated	48 (28)	45 (26)	
Class III/Contaminated	0 (0)	3 (2)	
Class IV/Dirty-Infected	0 (0)	1 (1)	
No Incision	1 (1)	0 (0)	
Temperature at Time of Procedure, median (IQR), °C	37 (36–37)	37 (36–37)	0.20
Actual Body Weight, median (IQR), kg	75 (63–88)	71 (61–88)	0.20
BMI, median (IQR), kg/m^2^	27 (24–31)	26 (23–31)	0.19
Classification of Hospital Stay			0.14
Inpatient	91 (54)	82 (48)	
Outpatient Invasive Procedure	37 (22)	55 (32)	
Observation	33 (19)	24 (14)	
Outpatient Surgical Center	9 (5)	9 (5)	
MRSA Nares Collection, n (%)	50 (29)	42 (25)	0.39
MRSA Nares Positive, n (%)	3 (6)	0 (0)	0.17
Surgical Irrigation Antibiotic Administered, n (%)	62 (36)	71 (42)	0.37
Penicillin + Other β-Lactam Allergy	8 (5)	10 (6)	0.81
Penicillin + Non-β-Lactam Allergy	56 (33)	41 (24)	0.09

IQR, interquartile range; BMI, body mass index; MRSA, methicillin-resistant *Staphylococcus aureus*.

*Other: surgery types < 5% in both groups. Surgery types included: vascular; colorectal; breast; plastic surgery; biliary tract; head and neck, clean-contaminated; thoracic; gastroduodenal; small intestine, non-obstructed; clean with risk factors or clean-contaminated; appendectomy for uncomplicated appendicitis; laparoscopic procedure, elective high-risk; head and neck, clean with placement of prosthesis; and small intestine, obstructed.

Secondary outcomes are listed in Table 2. There was no increase in documented allergic reactions or adverse drug reactions resulting from the intervention. Furthermore, no allergic reactions were suspected due to antibiotics. One case of *Clostridioides difficile* infection occurred in the pre-group. In the post-group, one case of blistering occurred secondary to intravenous vancomycin infiltration. All other adverse drug reactions were related to procedural sedation or had no identified cause.

**Table 2.** Results for secondary outcomes

Secondary Outcomes	Pre-Education Group (n = 170)	Post-Education Group (n = 170)	*P* Value
All-Cause Readmission within 90 Days, n (%)	27 (16)	14 (8)	0.04
All-Cause Mortality within 90 Days, n (%)	1 (1)	1 (1)	1.00
Surgical Site Infections (SSIs) Documented, n (%)	9 (5)	3 (2)	0.14
Superficial Incisional SSI	7 (4)	2 (1)	
Deep Incisional SSI	1 (1)	0 (0)	
Organ/Space Incisional SSI	1 (1)	1 (1)	
Acute Care Length of Stay, median (IQR), days	2 (1-4)	1 (1-3)	0.04
Documented True/Suspected Allergic Reaction During Admission, n (%)	2 (1)	1 (1)	1.00
Severe	0 (0)	1 (1)	
Non-Severe	2 (1)	0 (0)	
Adverse Drug Reaction During Admission, n (%)	3 (2)	5 (3)	0.72
Pharmacy Allergy Intervention, n (%)	10 (6)	19 (11)	0.12
Pre-Operative Guideline-Directed Dose Selection, n (%)	135 (79)	139 (82)	0.32

IQR, interquartile range.

In the post-education arm, a total of four SSIs occurred, three in patients who were readmitted (two superficial and one organ/space) and one in a patient seen in the emergency department without admission (superficial). Only one SSI had a positive culture. The patient had an ileostomy take-down procedure, and the pelvic surgical culture resulted in moderate *Escherichia coli* and *Klebsiella pneumonia*; the patient received vancomycin, which was not a first-line regimen for surgical prophylaxis for their procedure. The other patients with SSIs received appropriate regimens based on their surgery type and penicillin allergy reaction history.

## Discussion

There was a significant improvement in optimal preoperative antibiotic regimen utilization, with a 15% increase in utilization post-education. The educational intervention was associated with significant reductions in all-cause readmission within 90 days and acute care length of stay. These primary endpoint findings are attributed to increased understanding of true penicillin allergies while secondary endpoints may have been influenced by more preferred antibiotic use in surgical prophylaxis. Prior studies have shown clinical utility in performing pharmacist-led initiatives to remove false penicillin allergy labels.^7–9^ Our findings show agreement with previously published studies regarding our primary outcome. An increase in optimal regimen utilization was observed across most surgery types, with the only major exception being neurosurgery, indicating the results were not driven by a single group.

An area of interest moving forward from this pragmatic study is developing a long-term education strategy to ensure the progress made through this educational initiative is sustained as time passes and physician turnover occurs. Educational materials with strong evidence on the importance of reviewing allergy labels should be developed to further improve upon this research project. While strategies of allergy de-labeling were outside of this study’s scope and readily available resources, methods such as skin testing and oral antibiotic test dose challenges can be used to reduce allergy labels. Validated risk assessments of penicillin allergy labels have been shown to identify low-risk penicillin allergies that do not require formal allergy testing.^10^ These strategies may be considered as additional approaches to improving optimal antibiotic utilization.

Study strengths include the large sample size and similar baseline characteristics. Limitations of this study include the retrospective and single-center study design. Data could have been affected by medical record inaccuracies and data extraction errors. Continuing education throughout the year outside of the educational period may also have influenced the results of the study. A system-wide multidisciplinary initiative targeting a reduction in SSIs, specifically for colorectal, hysterectomy, and hip/knee replacement surgeries, occurred during this study, which may have been a confounding factor. To mitigate selection bias, list-generated patients were reviewed in order from most recent to furthest admission.

Overall, this educational campaign focusing on reasonable use of cefazolin for surgical prophylaxis in penicillin allergy-labeled patients may contribute to meaningful increases in use of optimal antibiotics while contributing to reductions in readmissions and acute care length of stay. Additional findings suggest a reduction in SSIs but were non-significant. Antibiotic allergies should be clarified to maximize use of optimal agents and improve patient outcome measures.

## Supporting information

Backus et al. supplementary material Backus et al. supplementary material

## Supplementary material

For supplementary material accompanying this paper visit http://doi.org/10.1017/ash.2024.428. click here to view supplementary material
