# Supplementary material for: Evaluation of antibiotic prescribing patterns for surgical prophylaxis before and after an educational campaign about penicillin allergies: a single-center, retrospective study
Source: Antimicrob Steward Healthc Epidemiol. 2024 Oct 10;4(1):e167. doi: 10.1017/ash.2024.428 (PMC11474901; doi:10.1017/ash.2024.428)
Supplement: Backus et al. supplementary material [file S2732494X24004285sup001.pdf]

WINK — *What I Need to Know*

Myth Busters
Cefazolin & Penicillin Allergy Labels

THE TRUTH ABOUT CEFAZOLIN & PENICILLIN ALLERGY LABELS

- Cefazolin is an injectable cephalosporin antibiotic, often used first-line for surgical prophylaxis
  - Penicillin allergy labels may prompt prescribing of 2nd- or 3rd-line antibiotics due to concerns for allergy cross-reactivity
- The latest data show the risk of penicillin allergy cross-reactivity to cefazolin is much lower than previously thought
  - Less than 5% of people with a history of penicillin allergy are actually found to be allergic when penicillin allergy tested
  - If 1,000 patients with an ambiguous penicillin allergy label received cefazolin, an allergic reaction would occur in only one
- Avoiding cefazolin due to a penicillin allergy label can mean patients do not get the safest and most effective care
  - Giving first-line antibiotics = less adverse events + improved patients outcomes
  - Patients who get second-line perioperative antibiotics can have a 50% increased risk for surgical site infections

DOES A PENICILLIN ALLERGY LABEL = ALWAYS AVOID CEFAZOLIN?

Myth Busted!... Many times patients with a penicillin allergy label can safely receive cefazolin!

- Complete allergy histories are essential for delivering the best care. In taking a history, beware patients can have more than one β-lactam drug allergy or an allergy to a cephalosporin but not a penicillin.
- A time to avoid cefazolin (unless otherwise instructed by the prescriber) is for patients with anaphylaxis caused by a penicillin
- Did you know? → Kaiser Permanente has removed all warnings within their system stating to avoid cephalosporins in the setting of penicillin allergy label, which includes patients with a history of penicillin-associated anaphylaxis… *It's true!*
- Structural side chains on penicillin and cephalosporin antibiotics have been found to be predictive for cross-reactivity potential
  - **Cefazolin does not share a similar structural side chain with any penicillin, other cephalosporin, or carbapenem!**

Chemical structures with side-chains circled, note cefazolin is different than penicillin or amoxicillin

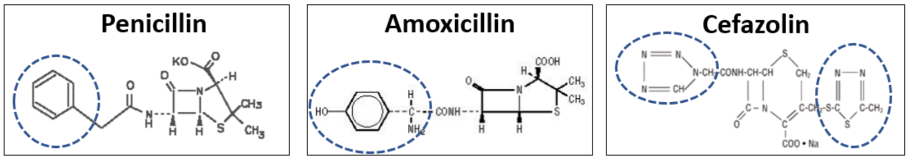

WHAT CAN WE DO?

1. **Collect A Complete Allergy History** → What antibiotic, onset/ type/ severity of reaction, who reported, timeframe occurred, and have they tolerated other beta-lactam agents such as amoxicillin-clavulanic acid (Augmentin®), piperacillin-tazobactam (Zosyn®), cephalexin (Keflex®), cefdinir (Omnicef®), cefuroxime (Ceftin®), ceftriaxone (Rocephin®), meropenem (Merrem®)?
   - Document within the electronic medical record under "Allergy tab" → Add Comments
2. **No History of Anaphylaxis or Severe Delayed/ Organ-Specific Reaction to a Penicillin?** → Consider using cefazolin
3. **History of Anaphylaxis or Severe Delayed/ Organ-Specific Reaction to a Penicillin?** → Consider avoiding cefazolin

Don't forget to document any new β-lactam allergy details in medical record!

RESOURCES & READINGS

1. Antimicrobial Prophylaxis in Surgery Guidelines
2. Khan DA, et al. *J Allergy Clin Immun*. 2022
3. Blumenthal KG et al. *CID*. 2018;66(3):329-36
4. Collins et al. *OFID*. 2021;9(1):ofab544
5. Macy, E. *J Allergy Clin Immun.* 2021
6. Zagursky R, et al. *J Allergy Clin Immun.* 2018
7. Lam et al. *Clin Infect Disease*. 2020
8. Liang et al. *J Allergy Clin Immun.* 2020
9. Chaudhry et al. *Pharmacy.* 2019;7(3):103
10. Sousa-Pinto B, et al. *JAMA Surgery. 2021*

Contact your antimicrobial stewardship pharmacist, pharmacy clinical coordinator, or infectious diseases consult service with questions.

The information presented here is summarized for simplicity. For full details refer to the resources and readings.
